# Interrater Reproducibility of Knee Movement Analyses during the Stance Phase: Use of Anatomical Landmark Calibration with a Rigid Marker Set

**DOI:** 10.1155/2013/692624

**Published:** 2013-10-03

**Authors:** Takashi Fukaya, Hirotaka Mutsuzaki, Yasuyoshi Wadano

**Affiliations:** ^1^Department of Physical Therapy, Faculty of Health Sciences, Tsukuba International University, 6-8-33 Manabe, Tsuchiura, Ibaraki 300-0051, Japan; ^2^Department of Orthopedic Surgery, Ibaraki Prefectural University of Health Sciences, 4669-2 Ami, Ami-machi, Ibaraki 300-0394, Japan

## Abstract

*Objective.* Measurements of knee joint movement in gait analysis may result in large errors caused by misplacement of reflective markers by the testers. To properly understand the measurement results, it is important to guarantee the reliability of the measurement method used for the purpose. The aim of this study was to confirm the interrater reproducibility of a measurement method with a rigid marker set (RMS). *Methods.* The study subjects were four healthy adults, and the testers were three physical therapists. The interrater reproducibility of the measurements was verified by using the coefficient of multiple correlations (CMCs) and the standard error of measurement (SEM). *Results.* The average CMCs values of 4 subjects in knee joint movement at the stance phase were greater than 0.8, and the average SEM values of 4 subjects in knee joint movement at the stance phase were also relatively good (maximum error: 2.42°). *Conclusion.* Based on these results, the measurement method with estimation of anatomical landmarks using the RMS can prevent misplacement during attachment of the reflective markers, as long as the testers have sufficient experience in attaching reflective markers.

## 1. Introduction

Gait analysis for musculoskeletal disorders and neuromuscular conditions (cerebral palsy, adult hemiplegia, and Parkinson's disease) has been widely applied both clinically and in research, and measurements using infrared reflective markers are an established technique for gait analysis [1]. In general, motion analysis based on a camera system is used to define a rigid body model in three-dimensional space. When the changes in joint movement are calculated by a rigid body model, the reflective markers are stuck on anatomical landmarks of each segment, and the changes in joint movement are calculated by the positions of the reflective markers. However, when a bone projection is used as an anatomical landmark, it has been reported that individual reflective markers stuck on the skin surface will move independently owing to modification of the soft tissue organization according to the shock in the early stance phase or the influence of muscle contraction [2]. While measurements of marker sets mounted on bone pins are comparatively accurate, the procedure is invasive and difficult to use in clinical settings [3, 4]. In addition, three markers mounted on rigid plates (rigid marker set (RMS)) affixed to the thigh and shank have been used to counter the problems associated with skin-mounted marker sets [5]. Since the infrared reflective markers are fixed to rigid plates in this method, the markers never move independently with deformation of the skin. However, the definition of the local segment system does not match the anatomical landmarks, and thus the method using rigid plates has associated difficulties for calculations of kinematics and kinetics data. Therefore, methods for estimating anatomical landmarks with at least three infrared reflective markers on rigid plates are used, but misplacement of the infrared reflective markers on anatomical landmarks by the testers is presumed to greatly affect the calculations of kinematics and kinetics data.

When the tester sticks reflective markers on anatomical landmarks identified by palpation, it is necessary to identify each anatomical landmark. Using a method for estimating anatomical landmarks with RMS containing three reflective markers attached to the thigh and shank, we examined the reproducibility of measurements within testers in a previous study [6] but did not evaluate the reproducibility of measurements between testers.

Measurement errors in gait analysis may be caused by misplacement of anatomical landmarks between testers. Therefore, to properly understand the measurement results, it is important to guarantee the reliability of the measurement method. The purpose of this study was to investigate the interrater reliability of knee movement analyses during the stance phase using the RMS. The hypothesis of this study is that the RMS has the potential to be valuable by accurately attaching to anatomical landmarks.

## 2. Methods

### 2.1. Study Design

**Figure 1:**
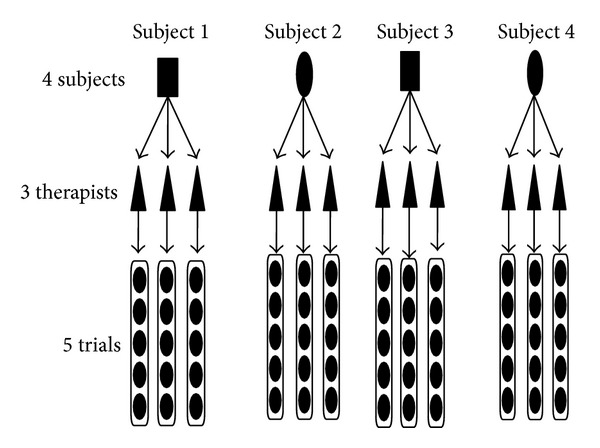
Study design and measurement procedure. After five trials, the infrared reflective markers were removed, and another tester then attached the infrared reflective markers to the same subject.

Each of three testers attached the infrared reflective markers to four subjects. All subjects were instructed to perform five trials of walking along a 10 m walkway after attachment of the infrared reflective markers by the first tester. The infrared reflective markers were removed after all five trials. The second tester then attached the infrared reflective markers to the same subject, and another five trials were performed as described above. The same procedure was repeated for the third tester. An overview of the study design is shown in Figure 1.

### 2.2. Subjects

The study subjects were four healthy adults (2 males and 2 females; mean age ± SD, 29.3 ± 6.1 years; mean height ± SD, 1.64 ± 0.13 m; mean mass ± SD, 61.7 ± 16.7 kg; mean BMI ± SD, 22.68 ± 2.53 kg/m^2^) who showed no evidence of orthopedic disease of the lower limbs or spine, no neurological impairment, and no limitations to the activities of daily life. All the subjects provided written informed consent prior to assessment. Ethical approval for the study was obtained from the Ibaraki Prefectural University of Health Sciences Ethics Committee.

### 2.3. Procedure

**Figure 2:**
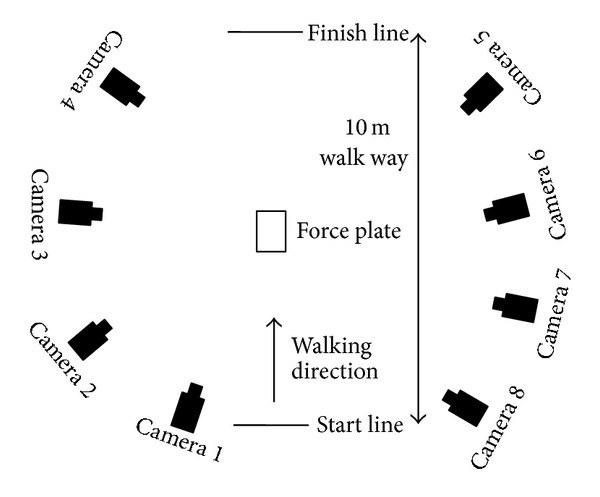
Schematic graph of the walking space and eight-camera system.

**Figure 3:**
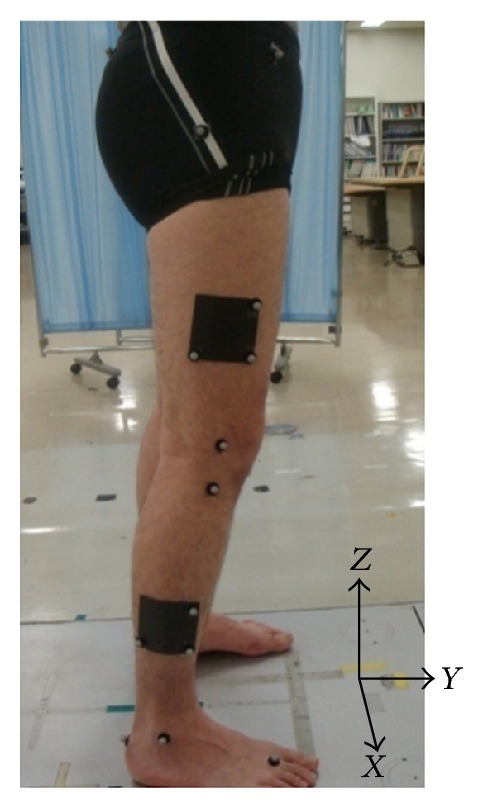
Rigid plates (black squares) with three reflective markers were attached to the thigh and shank.

A three-dimensional motion analysis system (Vicon, Oxford, UK) and a floor-mounted force plate (Kistler Instruments, Winterthur, Switzerland) such that each had a sampling rate of 200 Hz were used in this study (Figure 2). The testers were three physical therapists with more than 10 years of experience. Each subject performed one set of five trials for each of the three testers, comprising a total of 15 trials. The infrared reflective markers were removed from the subject after each set of five trials. The markers were attached directly with double-sided tape after confirmation of the anatomical landmarks by palpation. The markers were directly placed over the following anatomical landmarks: bilateral anterior and posterior superior iliac spines; unilateral greater trochanter; lateral and medial femoral epicondyles; lateral and medial tibial condyles; lateral and medial malleoli; calcaneus; and top of the foot at the base of the second metatarsal. In addition, the RMS plates with three attached reflective markers were placed on the lateral side of the thigh at the same line between the greater trochanter and the lateral femoral epicondyles and shank at the same line between the lateral femoral epicondyles and the lateral malleoli (Figure 3). The subjects walked barefoot along a 10 m walkway at their self-selected habitual speeds and were directed to step onto the force plate with the right lower limb. The number of trials was five, with rest time of 30 sec between the trials. After attachment of the markers, decisions were made for the relative positions of the anatomical landmarks for the two rigid plates with the RMS, based on a single static calibration to estimate the anatomical landmarks of the thigh and shank from the RMS. The anatomical landmarks used for the thigh and shank clusters were the greater trochanter, lateral and medial femoral epicondyles, lateral and medial tibial condyles, and lateral and medial malleoli. The estimation of the anatomical landmarks from the RMS was performed using mathematical software (Vicon Bodybuilder; Vicon).

### 2.4. Data Analysis

Foot-strike (FS) and toe-off (TO) were determined using the force plate data, and the corresponding frame number was identified in the kinematics data. The kinematics data were normalized to the 100% stance phase (FS to TO = 100%) using spline interpolation. To calculate the kinematics data, the local coordinate systems of the thigh and shank were defined in the three-dimensional position by the anatomical landmarks of the thigh and shank estimated from the RMS, and the knee joint angles during the stance phase were calculated using the joint coordinate system (JCS) approach described by Grood and Suntay [7]. The global coordinate system was defined as follows: *X*-axis was lateral, *Y*-axis was anterior, and *Z*-axis was vertical (Figure 3). The coordinate systems for the thigh (*T*) and shank (*S*) were defined as follows:

 
*T*_*x*_: vector directed laterally from the medial to lateral femoral epicondyle;
 
*T*_*y*_: cross-product of a vector directed anteriorly from the knee joint center (midpoint between the medial and lateral femoral epicondyles) to the greater trochanter and *T*_*x*_;
 
*T*_*z*_: cross-product of vectors *T*_*x*_ and *T*_*y*_;
 
*S*_*x*_: cross-product of vectors *S*_*y*_ and *S*_*z*_;
 
*S*_*y*_: cross-product of *S*_*z*_ and a vector directed anteriorly from the medial to lateral tibial condyle;
 
*S*_*z*_: vector directed from the ankle joint center (midpoint between the lateral and medial malleoli) to the midpoint between the medial and lateral tibial condyles.

To calculate the knee joint angles, two axes of the JCS were embedded in the two segments whose relative motion was to be described. The two vectors were the *T*_*x*_ vector of the thigh coordinate system and the *S*_*z*_ vector of the shank coordinate system. The third axis was called the floating axis and was the common perpendicular to both *T*_*x*_ and *S*_*z*_. Flexion-extension occurred about the *T*_*x*_ axis. The flexion-extension angle, *α*, was obtained by the angle between *T*_*y*_ and the floating axis, and flexion was positive when extension was negative. External-internal rotation occurred about the *S*_*z*_ axis. The external-internal angle, *γ*, was obtained by the angle between *S*_*y*_ and the floating axis, and external was positive when internal was negative. Abduction-adduction occurred about the floating axis. The abduction-adduction angle, *β*, was obtained by the value in which *π*/2 was pulled from *δ*, defined as the angle between *T*_*x*_ and the *S*_*z*_ axis. Abduction was positive when adduction was negative.

### 2.5. Statistical Analysis

To check the reproducibility of the waveform data for the knee joint angle for each subject between testers, the coefficient of multiple correlations (CMCs) was computed according to a previously described method [8] as an index of relative reliability using the following formulas:

(1) $$\begin{array}{c} \text{CMC}=\sqrt{1-\frac{\sum_{i=1}^{M}\sum_{j=1}^{N}\sum_{t=1}^{T}{\left(Y_{ijt}-{\overset{-}{Y}}_{t}\right)}^{2}/T\left(\text{MN}-1\right)}{\sum_{i=1}^{M}\sum_{j=1}^{N}\sum_{t=1}^{T}{\left(Y_{ijt}-\overset{-}{Y}\right)}^{2}/\left(\text{MNT}-1\right)},} \end{array}$$

where *M* is the number of test days, *N* is the number of the trials, *T* is the number of the data points, ${\overset{-}{Y}}_{t}$ is the average at time point *t* over MN gait cycles, and $\overset{-}{Y}$ is the mean over time. Furthermore, in each subject's stance phase, to check how much error of the knee joint angle appeared at the time when the SD was the largest, the standard error of measurement (SEM) was computed as an index of quantitative reliability [9] using the following formulas:

(2) SEM=s1−r,

where *s* is the standard deviation and *r* is the coefficient of correlations. In this study, the calculation of SEM used the coefficient of correlations without ICC for an index of relative reliability with the time-dependent changes in the knee joint angles.

## 3. Results

**Figure 4:**
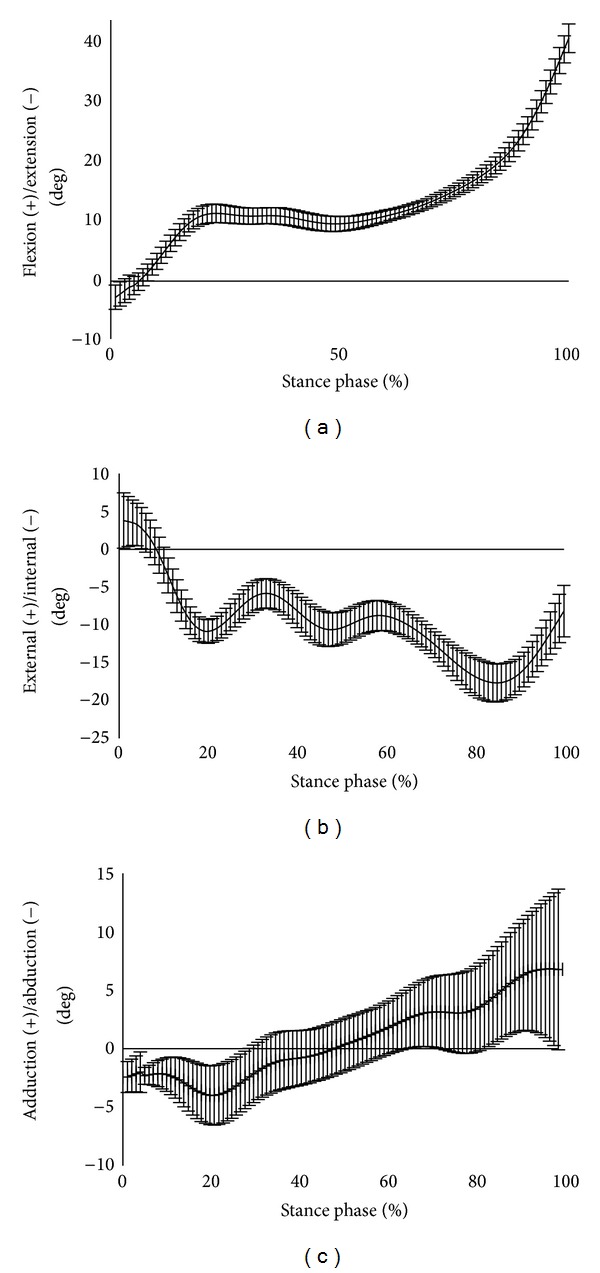
Representative data for the joint angle of Subject 3: (a) flexion-extension; (b) external-internal rotation; (c) abduction-adduction. The vertical bars show the SD.

As representative data, Figure 4 shows the mean and SD values of the knee joint angle changes in the stance phase of Subject 3 evaluated by the three testers. Moreover, Table 1 shows the CMC values between testers for each of the subjects. The CMC values for reliability ranged from 0.91 to 0.94 for flexion-extension, 0.94 to 0.97 for external-internal rotation, and 0.77 to 0.92 for abduction-adduction.  Table 2 shows the SEM results. The SEM values for reliability ranged from 0.68° to 1.13° for flexion-extension, 0.78° to 1.60° for external-internal rotation, and 1.43° to 3.33° for abduction-adduction.

## 4. Discussion

This study was carried out to verify the measurement reproducibility between testers in gait analysis. The local coordinate system of each segment in three-dimensional motion of the knee joint is created by anatomical landmarks of the thigh and shank, and the results for knee joint motion are greatly influenced by their positional relationship. Previously, we examined the reproducibility of measurements between sessions by one tester using the same method as in this study, and the CMC values showed very good results of ≥0.8 for the three-dimensional motion of the knee joint [6]. In general, the measurement errors between testers are considered to be greater than the measurement errors between sessions and within testers [10], and the results for the intertester reproducibility of measurements in the present study were also >0.8. These findings are thought to rise because all three testers were physical therapists with more than 10 years of clinical experience and the anatomical landmarks were relatively easy to palpate from the skin surface. The young inexperienced testers might get lower reliability of the same measurements.

In this study, the reproducibility between testers showed relatively good results. However, the error in the knee joint angle for all subjects showed a tendency to increase at the time of FS or TO. The reason for this was thought to be that the RMS of the thigh was attached to the surface of the skin overlying great muscle contraction in the early stance, leading to errors in the anatomical landmarks estimated by the RMS. In addition, the force required to kick the floor by the triceps muscles in the late stance phase demonstrates forward propulsion [11]. Therefore, errors from the RMS on the shank occurred. In measurement methods using infrared reflective markers, the problem of measurement accuracy with regard to skin movement artifacts has previously been discussed [12] although a resolution remains to be obtained [5]. We compared the measurement errors between the RMS and the point cluster technique (PCT [13]), which is able to capture relatively accurate three-dimensional motion of the knee joint using infrared reflective markers. The knee movements of flexion-extension and abduction-adduction produced results with small measurement errors by the RMS and PCT [14]. Considering the results of the present and previous studies, since the measurement method for estimating anatomical landmarks with the RMS involves a small number of infrared reflective markers and these markers can be attached to certain positions for easy identification, even by different testers, it may be relatively easy to use this measurement method in clinical practice.

The limitation of this study may be follows. In this study, all three testers were physical therapists, each with more than 10 years of experience. Therefore, it should be pointed out that relatively inexperienced testers may produce different results. Another limitation of this study was that the number of subjects was too few. We are considering application of the measurement method with the RMS to diseases such as osteoarthritis of the knee or anterior cruciate ligament injury in the future.

## 5. Conclusions

The findings of the present study appear to support clinical use of the RMS, since the method was able to prevent misplacement during attachment of the markers to anatomical landmarks that could be easily palpated by well-trained testers. In addition, the interrater reproducibility for use of the RMS was confirmed in this study.

## Figures and Tables

**Table 1:** Coefficients of multiple correlations of the knee angles in the stance phase of each subject.

	Flex.-ext.	Ext.-int.	Add.-abd.
Subject 1	0.94	0.95	0.81
Subject 2	0.93	0.94	0.92
Subject 3	0.92	0.95	0.77
Subject 4	0.91	0.97	0.78

Ave.	0.93	0.95	0.82

**Table 2:** Standard errors of measurements of the knee angle in the stance phase of each subject.

	Flex.-ext.	Ext.-int.	Add.-abd.
Subject 1	0.90°	1.60°	3.01°
Subject 2	0.72°	1.06°	1.43°
Subject 3	0.68°	0.78°	3.33°
Subject 4	1.13°	0.87°	1.93°

Ave.	0.86°	1.08°	2.42°
